# Honey Bee Alarm Pheromone Mediates Communication in Plant–Pollinator–Predator Interactions

**DOI:** 10.3390/insects10100366

**Published:** 2019-10-21

**Authors:** Zhengwei Wang, Ken Tan

**Affiliations:** 1 CAS Key Laboratory of Tropical Forest Ecology, Xishuangbanna Tropical Botanical Garden, Chinese Academy of Sciences, Kunming 650000, China; 2 Center for Plant Ecology, Core Botanical Gardens, Chinese Academy of Sciences, Mengla 666303, China

**Keywords:** olfaction, isopentyl acetate, honest signal, I-see-you signal, mimicry

## Abstract

Honey bees play a crucial role in pollination, and in performing this critical function, face numerous threats from predators and parasites during foraging and homing trips. Back in the nest, their defensive behavior drives some individuals to sacrifice themselves while fighting intruders with their stingers or mandibles. During these intense conflicts, bees release alarm pheromone to rapidly communicate with other nest mates about the present danger. However, we still know little about why and how alarm pheromone is used in plant–pollinator–predator interactions. Here, we review the history of previously detected bee alarm pheromones and the current state of the chemical analyses. More new components and functions have been confirmed in honey bee alarm pheromone. Then, we ask how important the alarm pheromones are in intra- and/or inter-species communication. Some plants even adopt mimicry systems to attract either the pollinators themselves or their predators for pollination via alarm pheromone. Pheromones are honest signals that evolved in one species and can be one of the main driving factors affecting co-evolution in plant–pollinator–predator interactions. Our review intends to stimulate new studies on the neuronal, molecular, behavioral, and evolutionary levels in order to understand how alarm pheromone mediates communication in plant–pollinator–predator interactions.

Honey bees play a crucial role in ecosystems via pollination [1,2]. However, while performing this critical ecosystem service, honey bees encounter multiple predation threats, such as weaver ants, spiders, mantises, hornets, and birds during foraging or even at their own hive [3,4]. In order to protect the colony and the individual from predation threats, honey bees use alarm pheromone to rapidly communicate with their hive mates at the entrance of the hive and to alert foragers about dangerous foraging sites [4]. During foraging, honey bees make decisions based on potential costs and benefits and may choose a lower nectar concentration food source over another source with a fluctuating concentration [5]. They also adjust their foraging load to compensate for foraging risks [5] using information from alarm pheromone, which may also function at the level of predation risks. These phenomena are all a part of co-evolutionary relations between plants, pollinators, and predators.

Honey bees are social insects that are well known for their symbolic and olfactory communication [6]. Although it is still unknown exactly why honey bees evolved with a barbed stinger [7], the release of the sting produces alarm pheromone and certainly can be helpful at the entrance of the hive, where this sacrificial defensive behavior can be seen as a chemical marking which orients other guard bees to join in the defense.

Alarm pheromones appear to be the second most commonly produced class of chemical signals used by insects for rapid communication, after sex pheromones [8]. In the honey bee community, alarm pheromone is a highly efficient signal used for alerting and recruiting, leading to a more efficient colony defense. In this context, these chemical signals serve multiple functions. A group of elder honey bees, called guard bees, patrol the hive entrance. These guards are also specialized for the production of alarm pheromone, which they release to recruit nest mates from the interior of the colony when they encounter danger [9,10]. Beekeepers are well acquainted with the banana-like odor released by stressed bees. Often, one bee sting is followed by additional stings from other guard bees unless the intruder rapidly moves away from the aroused colony. Beekeepers also use smoke to sedate aroused bees [8].

In this paper, we review the history of the study of honey bee alarm pheromone. Then, we focus on the communication functions of alarm pheromone on intra-specific or inter-specific species between *Apis* and other predators. We examine how plants could benefit from alarm pheromones in terms of pollination. Finally, we posit potential implications of stated research and offer directions for future areas of research on honey bee alarm pheromone.

## 1. The History of Detecting Bee Alarm Pheromone and Its Components

The first recording that a honey bee alarm signal could act in order to alert honey bee workers dates back to the early 17th century (Bulter, 1609) cited in [8]. The signal was proposed to be an odorant, and it was suggested that when bees get close to another honey bee worker’s sting, they would change their behavior from calm to aggressive (Huber, 1814) cited in [8]. These chemicals were later identified as the honey bee alarm pheromone and found to be produced in the Koshevnikov gland, associated with the sting apparatus as well as in the mandibular gland [9,11].

Isopentyl acetate was the first identified alarm pheromone in bees associated with the sting which showed biological activity [9]. More than 20 additional volatile aliphatic and aromatic active compounds of low molecular weight have been isolated and identified in the alarm pheromone blend [12,13,14]. As scientific works expanded to other species, an oil-like compound, (Z)-11-eicosen-1-ol, was thought to play an essential role in the alarm pheromone of *A. cerana* [15]. Shearer and Boch [11] then reported that when 2-heptanone was isolated from the honey bee mandibular gland, the compound also produced alarm activity.

Different components of the pheromone blend in honey bees induce different defensive behaviors, such as alarming (isopentyl acetate, (Z)-11-eicosen-1-ol), flight activity (benzyl acetate), recruitment (1-butanol, 1-octanol, hexyl acetate), and some of these chemical components play multiple roles, such as recruitment to a food source, alerting returning bees, and repelling foraging activity (1-hexanol, butyl acetate, isopentyl acetate, 2-nonanol) [16]. Since the pioneering works of Koeniger, et al. [17], an increasing number of chemicals have been identified and their functions clarified (Table 1).

## 2. Bee Alarm Pheromones Mediate Intra- and Inter-Species Communication

Mandibular and Koshevnikov glands associated with the sting apparatus are the main alarm pheromone producing organs in the *Apis* genus. Even though different bee species inhabit different environments with unique conditions, most of the bee species still secrete isopentyl acetate as the main compound [9,11] (Table 1). Some species-specific compounds have been identified in different bee species. Here, we mainly focus on the honey bees’ (*Apis* genus) use of alarm pheromone to rapidly communicate within and between species.

### 2.1. Intra-Species Communication

Honey bees produce alarm pheromone which initiates an alert state in other nest mates, making them ready for aggression and defense. The major component, isopentyl acetate, found in most of the honey bee species, can elicit stinging when encountering intruders, attracting other nest mates to join in defense, and repelling foragers at flowers or artificial food sources in *A. mellifera* [17,25]. The other oil-like component, (Z)-11-eicosen-1-ol, which has been identified in both *A. cerana* and *A. mellifera*, can trigger stinging [15,19].

Some other components, such as 1-hexanol, octyl acetate, butyl acetate,1-butanol, 1-octanol, hexyl acetate, and 2-nonanol found in the *A. mellifera* alarm pheromone may not elicit stinging but could help to recruit other nest mates to attend to defense activity [9,10,19,23].

There are some compounds which had been identified in the *A. mellifera* alarm pheromone but were later found to be alarm pheromone carriers (eicosenol) or have no identified functions [19].

Another component different from those identified in the Koshevnikov gland and found in the mandibular gland is 2-heptanone. This compound also elicits alarm pheromone behaviors [11]. Some studies have shown that it acts as a repellent or does not elicit any reaction in guard bees [26], and the most probable function may be to serve as a forage-marking pheromone.

Few studies have focused on alarm pheromone identification in species other than *A. mellifera*. This is mainly because of the restricted distribution of most of the other bee species. Morse, et al. [22] quantified the amount of isopentyl acetate in *A. mellifera*, *A. cerana* (addressed as *A. indica*, and should now be *A. cerana indica*), *A. dorsata*, and *A.florea*. It was found that the alarm compounds varied based on body size. Koeniger, et al. [17] identified and compared alarm pheromone from *A. mellifera*, *A.cerana*, *A. florea,* and *A. dorsata* in regards to both components and functions (Table 1). Blum, et al. [20] studied alarm pheromone in two giant honey bee species (*A. dorsata* and *A. laboriosa*), but only mentioned one more new component, gamma-octanoic lactone in *A. laboriosa*. With the benefit of easily accessing all five of these honey bee species and the development of better chemical analyses and tools, we reanalyzed the alarm pheromone of *A. cerana*, *A. florea,* and *A. dorsata*. More sophisticated Gas Chromatography (GC) columns and absorbing fibers are now available, allowing an increase in efficiency of compound isolation and identification. Now, new components such as benzyl alcohol, (E)-oct-2-en-1-ol, (E)-oct-2-en-1-ol acetate, phenethyl acetate, and (E)-2-decen-1-yl have been identified in *A. cerana*. In fact, benzyl acetate has been identified as the main component of the *A. cerana* alarm pheromone, in quantities similar to that of isopentyl acetate in *A. mellifera*. Benzyl acetate may alert returning bees at the hive entrance during threatening attacks, and may repel foragers at the feeder [18]. (E)-dec-2-en-1-yl acetate has been shown to exist in the alarm pheromone of all three species [18]. The fact that these two compounds have not been identified in previous studies may also be because of their variable quantification in guards and foragers (2–3-fold higher) [18].

Gamma-octanoic lactone was further discovered in the alarm pheromone of the *A. dorsata* mandibular gland rather than in the Koshevnikov gland [18]. This was discovered after altering the experimental methods. Previously, only the wings of *A. dorsata* were clipped, causing the giant honey bee to secrete both gamma-octanoic lactone from their mandibular gland and isopentyl acetate, (E)-2-dencen-1-yl acetate, octyl acetate and other chemicals from the Koshevnikov gland [12]. Through direct gland dissection, it was definitively determined that gamma-octanoic lactone in *A. dorsata* comes from the mandibular gland.

Realistic doses of both single chemicals and mixtures of gamma-octanoic lactone, isopentyl acetate and (E)-2-decen-1-yl acetate were proven to be highly repellent when *A. dorsata* bees foraged at food sources [10,12] (Table 1). 

Similarly, as found in *A. mellifera*, some compounds such as isopentyl propionate, octyl acetate and 3-methyl-1-butanol showed no significant alarm function in *A. dorsata* [16]. Whether they work as carriers or in expanding the effects of other active compounds still needs clarification.

### 2.2. Inter-Species Communication

Some compounds of honey bee alarm pheromone, such as isopentyl acetate, octyl acetate, and benzyl acetate, were found in all four *Apis* species (*A. mellifera*, *A. cerana*, *A. dorsata,* and *A. florea*). Isopentyl acetate is considered to be one of the primary chemicals existing in every *Apis* genus, even though there are still no reports for *A. laboriosa*, or any of the other recognized honey bee species (the nine bee species system [27] or 11 bee species [28]). The compound 3-methyl-1-butanol has been commonly found in the *A. mellifera* and the *A. dorsata* alarm pheromone, while (E)-2-decen-1-butanol has been identified in *A. cerana*, *A. dorsata* and *A. florea*. The compound 1-octanol was found in *A. mellifera* and *A. dorsata*. Gamma-octanoic lactone exists in the two giant honey bee species [18].

Do honey bees eavesdrop from different honey bee species? Since bee species share some common compounds, yet also have some different compounds between their alarm pheromones, how efficient is the communication between intra- and inter-species using alarm pheromones? Goodale and Nieh [29] tested this eavesdropping system with bumble bees and honey bees, and found no evidence for such eavesdropping. Specifically, bumble bees showed no avoidance of honey bee alarm pheromone left at a food source. Our recent work tested this question with three honey bee species, *A. cerana*, *A. mellifera,* and *A. dorsata*, which revealed that *A. cerana* foragers avoid both intra-specific and inter-specific alarm pheromones when foraging at food sources. This is not only because *A. cerana* recognized isopentyl acetate, which exists in all three honey bee species, but because *A. cerana* also responded aversively to gamma-octanoic lactone which only exists in the giant honey bee species [30].

## 3. Bee Alarm Pheromones Mediate Communication between Pollinators and Predators/Parasites

The communication between predators and pollinators is related to pollinators’ defensive fighting. The fighting organs of the *Apis* species produce alarm pheromone when pollinators bite with their mandibles or sting with their stingers. Although commonly comprising a mixture of several compounds, alarm pheromone tends to be less specialized than other kinds of pheromones, and few are species-specific [31]. This relative non-specificity may be an advantage to all *Apis* species that are able to detect alarm signals of other species, sharing vulnerability to a common threat (described above, [30]), but this could also be a disadvantage if these olfactory signals are eavesdropped upon by a predator [8].

Honey bees face heavy predation threats when foraging among flowers and they also suffer from predators or parasites inside the hive because of the highly attractive nature of their food store [3]. Ants, birds, hornets, and the praying mantis are all highly harmful predators to honey bee colonies [3,4,32]. In addition mites, wax moths, and small hive beetles are important parasites both at the level of the individual and the colony [33,34,35].

### 3.1. Bee Alarm Pheromones Used in Communication between Pollinators and Predators—Ants

Ambushing ants preying on honey bees underneath flowers is a common condition in tropical areas [4]. Predators can influence pollinator behavior [36], which also drives pollinators to evolve social communication signals or to eavesdrop on predator signals [29,37] in order to better balance the trade-off between predation risks and floral rewards [38,39]. Evidence for this argument comes from the finding that honey bees avoid flowers with dead bodies, trail pheromones, or alarm pheromones from other bee species [4,40].

The tug-of-war between ants and bees results in ants not always congregating in large numbers together under a flower inflorescence or young twigs. Instead, only a few ant workers (1–2 ants) lie in ambush under one of the inflorescences of *Calliandra haematocephala*; the other ants rest at twigs within a 20–30 cm distance to the flowers. Only when the pioneer ant grabs the bee and causes it to release alarm pheromone (banana-like smell to our human nose) will the other ants then rush to the flower to catch the struggling bee [4,12]. We then asked whether ants would use honey bee alarm pheromone as a kairomone to locate their prey. Our experiments applying a synthetic mixture of the *A. dorsata* alarm pheromone and individual compounds to bee dummies proved weaver ants could sense and make use of bee alarm to aggregate for joint hunting (Wang et al., in preparation).

### 3.2. Bee Alarm Pheromones Used in Communication between Pollinators and Predators—Hornets

Hornets hunt for honey bees on flowers [41,42]. The hornet’s rapid colony development and strength was found to depend highly on their ability to feed on honey bees, and in turn increased the chances of more attacks both during pollination and at the entrance of the hive [41]. The honey bee species *A. cerana* has evolved a specific abdomen shaking behavior, called the I-see-you signal, an honest signal which repels *Vespa velutina* hornets hovering at the bee hive entrance [32,43]. If the hornet gets too close to the bee colony, guard bees form a heat ball to bake and smother the intruder to death [44,45,46,47].

Olfactory information used between hornets and honey bees is also an interesting phenomenon. When the hornets first find a honey bee colony, they leave chemicals from the van der Vecht gland to mark the location. Interestingly, honey bees can detect it and try to remove it [44]. Even while forming the heat balls, both honey bees and hornets release alarm pheromones along with attacking each other with their stingers. The chemicals produced from the heat ball have been identified as honey bee alarm pheromone compounds: isopentyl acetate, octyl acetate, (E)-2-decen-1-yl acetate and benzyl acetate [18]; and from the hornet alarm pheromone compounds: 2-heptanone, 2-nonanone, and 2-undecanone [48]. The Asian honey bee shows high electrophysiological responses to hornet alarm pheromone. Their guards are also easily recruited to attack [49]. So far, we still do not know how these hornet predators use honey bee alarm pheromone to trigger attacking or repelling in front of the bee hive. Another predator case is the beewolf, *Philanthus triangulum*, which can locate honey bee prey via honey bee olfactory signals [50].

### 3.3. Bee Alarm Pheromones Used in Communication between Pollinators and Parasites

Parasites in the bee hive, such as mites, wax moths and small hive beetles, harm the apicultural industry around the world [51,52,53]. Whether olfactory cues of bee signals can be eavesdropped upon by these parasites has drawn the attention of researchers [51]. Phoretic mites, *Neocypholaelaps indica*, highly infest the inflorescences of *Pachysandra axillaris* and its pollinators, *A. cerana* [54]. These observations may indicate that the parasites could make use of the alarm pheromone of bees for their dispersion during different stages of their life cycle. 

Low doses of alarm pheromone (isopentyl acetate, 2-heptanone) left at the hive entrance led to a high electro–antenno graphic detection (EAD) response of the small hive beetle, *Aethina tumida*. Isopentyl acetate alone is sufficient to attract the beetles [55]. We also tested whether wax moths use the alarm pheromone of honey bees as a signal to locate bee hives for invasion, and found that the wax moth is able to sense alarm pheromone through antennal responses, but they did not show avoidance behavior, possibly because wax moths invade bee colonies at night and have few chances to be attacked by guard bees and thus display no behavioral response to bee alarm pheromone [35].

## 4. Potential Ecological Effects

Both visual and olfactory stimuli play crucial roles in plant–pollinator mutualisms. Predators may hover or sit-and-wait at the flowers when preying on pollinators. These conditions may deter pollinator visits, thereby reducing fruit and seed setting [40,56]. On the other hand, plants may attract both predators and prey as pollinators, or predators only, for pollination by mimicking the cues of prey. Below, we focus on these two extreme cases in which plants mimic honey bee alarm pheromone either to attract honey bees only, or predators only for pollination. A context-dependent learning hypothesis was raised as an explanation for these two paradoxical conflicting phenomena.

### 4.1. Bee Alarm Pheromone Mimicry in Plants to Attract Honey Bees for Pollination

Risk related cues can deter the pollinator from approaching [40,57], however, more and more plant species have been studied whose flower compounds contain honey bee alarm pheromone compounds or similarly structured compounds. These various compounds have been found to be highly attractive to honey bees for pollination. However, most of the studies showing that flowers contained benzyl acetate and 3-methyl-1-butanol did not mention pollinators. According to our recent study, benzyl acetate could be a main compound in the pheromone of the Asian honey bee, *A. cerana* [18]. One of our collaborative partners found that winter flowering plants, such as *Pachysandra axillaris*, provided a pollen reward for *A. cerana*. The local bees showed more preference to this flower than other flowering plants in the same season. The most interesting finding was that benzyl acetate made up 95% of the floral volatiles in *P. axillaris* [54]. As reported, benzyl acetate was commonly found in several plant species [58]. So far, we still do not fully understand how much this alarm pheromone compound helps these plants to attract honey bees for pollination. Our current hypothesis suggests that this form of alarm pheromone mimicry is likely a different strategy to attract bees for pollination (the attracting threshold should overcome the innate repelling) than flower mimicry using honey bee queen pheromone (3-hydroxyoctanoic acid and 10-hydroxy-(E)-2-decenoic acid; only innate attracting) [59].

### 4.2. Bee Alarm Pheromone Mimicry in Plants to Attract Predators for Pollination

Orchids attract wasps by mimicking female sex pheromones [60,61] or green-leaf volatiles [62]. Another extreme case is to mimic the prey’ s alarm cues to increase pollination by its predators [21]. Based on previous GC-MS work, five compounds were identified from the orchid, *Dendrobium sinense*, from Hainan, China [21]. Previous work has found that benzyl acetate and benzyl alcohol are common volatiles in plants [58], leading to the conclusion that only octadecan-1-ol, eicosan-1-ol, and (Z)-11-eicosen-1-ol are flower odors mimicking the compounds released by *A. cerana* [21]. GC-MS recordings uncovered a benzyl acetate spike in *A. cerana* alarm pheromone. Electrophysiological responses of hornet antennae were tested against these floral volatiles using EAD, proving that all five compounds are EAD-active chemicals for hornet detection [21]. Hornet EAD recordings showed even higher electrophysiological responses to benzyl acetate and benzyl alcohol than to octadecan-1-ol, eicosan-1-ol, and (Z)-11-eicosen-1-ol [21]. Recently, Wen et al. confirmed that both benzyl acetate and benzyl alcohol were found in Asian honey bee alarm pheromone, and benzyl acetate affected the flight trajectory of returning bees.

### 4.3. Context-Dependent Learning Hypothesis

The foragers prefer to visit flowers with conspecific mates [63], which indicates that conspecific mate foraging activity could act as a cue. At the same time, foragers may leave trail pheromone or other marking pheromones to help conspecific mates to locate food sources [64,65]. Even if the flower contains alarm pheromone, which would originally be considered a repellent to pollinators, the alarm pheromone or predator cues can be discriminated in a context-dependent manner by bees. This means that if they are combined with a sugar reward, bees may learn to treat them as attractants [66]. Bees display a high behavioral plasticity and can learn alarm pheromone compounds in an appetitive context [67,68,69,70], even though they may display unexpected generalization to other odorants after learning these compounds [70]. This appetitive learning ability could explain why some flowers are able to attract both predators and pollinators, or stimulate predators to hover around pollinator foraging sites without deterring these pollinators [21,42]. It could be the reason why some flowers without a nectar reward are highly attractive for hornets as pollinators [21]. Particularly during the nectar dearth season, like winter in Kunming (25°8’48.9” N and 102°44’41.2” E), honey bees could be highly attracted to pollinate plants which contain alarm pheromone (benzyl acetate at levels up to 95%) [54]. This effect could explain why benzyl acetate is common in 38 families of studied plants and the dominant chemical, benzyl alcohol, occurs in 56% of their studied plants [58]. A high dose of alarm pheromone produced by plants may thus act as an olfactory cue which attracts a few scout bees occasionally without a repelling effect. Bees then learn to associate the alarm pheromone in the context of a reward and the foraging activity of these few bees may itself become a marker which will eventually attract more conspecific mates for pollination.

## 5. Potential Implications and Future Perspectives

The specific neuronal mechanisms in the honey bee brain that register signals of fear and safety still need investigation. Previous studies showed that pheromone compounds (including alarm pheromone) induce neural activity in the bee brain, both in the primary olfactory center, the antennal lobe [71,72] and the lateral horn [73]. In some cases, different pheromones like brood and queen pheromones were shown to be processed by different pathways [71]. As reviewed above, more than 20 alarm compounds have been identified. One study showed that each individual component of the alarm pheromone, such as isopentyl acetate, activates a different combination of antennal lobe glomeruli, but predicting which ones are activated by the full alarm pheromone blend is a complex task [72]. In addition, we still cannot segregate neural activity related to each compound’s chemical structure from that related to its pheromonal function [71,73]. Beyond the processing of individual pheromones in the brain, it is interesting to ask how do bees evaluate the trade-offs between foraging risks, individual survival and food reward? How do different concentrations of alarm pheromone compound or different ratios of the alarm pheromone blend encode different risk levels? In which regions of the brain are these signals received, combined and processed?

How do animals use the information and how do these signals drive the evolution of plant–pollinator–predator systems? Honey bee alarm pheromone evolved in different honey bee species, and the high overlap in the guiding function of the same chemicals may reflect a common evolutionary basis. What might be the evolutionary advantage to share similar chemicals for similar threats? Are there additional factors involved in the plants’ mimicking of alarm pheromone components besides the potential beneficial effect on pollination? A systematic study on plant–pollinator–predator interactions is needed for better understanding of the evolution of the signals used in these communication systems. A better understanding of these complex conditions may help to apply gene editing methods for the enhancement of pollination efficiency in some crop species.

## Figures and Tables

**Table 1:** Summary of chemical compounds in the alarm pheromone of different honey bee species in the *Apis* genus.

Compound	CAS Number	Species					Function to Bees	Reference
		*A. mellifera*	*A. cerana*	*A. dorsata*	*A. florea*	*A. laboriosa*		
**Alcohols** (Koshevnikov gland)								
1-Hexanol	111-27-3	+					Attracts recruits	[10]
1-Butanol	71-36-3	+					Attracts recruits	[10]
1-Octanol	111-87-5	+		+			Attracts recruits	[10,12]
2-Methyl-1-butanol	137-32-6	+					NA	[16]
3-Methyl-1-butanol	123-51-3	+	+	+			No repels foraging	[12,16,18]
2-Nonanol	628-99-9	+	+				Attracts recruits	[10,19]
(Z)-11-eicosen-1-ol	68760-58-7	+	+				Elicits stinging, Elicits attraction	[15,19]
Eicosenol	62442-62-0	+	+				Carrier of other active alarm pheromones	[19]
Farnesol	4602-84-0			+			NA	[20]
Benzyl alcohol	100-51-6		+				NA	[18,21]
(E)-oct-2-en-1-ol	18409-17-1		+				NA	[18]
(E)-2-decen-1-yl	18409-18-2		+				NA	[18]
1-Octadecanol	112-92-5		+				NA	[21]
**Esters** (Koshevnikov gland)								
Isopentyl acetate	123-92-2	+	+	+	+		Elicits stinging, Attracts recruits; Repels foraging	[9,12,17,22]
Octyl acetate	112-14-1	+	+	+	+		Elicits attraction; No repels foraging	[12,16,18,19]
Butyl acetate	123-86-4	+					Attracts recruits	[23]
Hexyl acetate	142-92-7	+		+			Attracts recruits	[9]
(E)-2-decen-1-yl acetate	19487-61-7		+	+	+		Extends the duration of other alarm components; Repels foraging	[12,17,18,24]
Benzyl acetate	140-11-4	+	+	+	+		Alerts returning bees; repel foraging	[18,23]
decyl acetate	112-17-4			+			NA	[20]
undecyl acetate	1731-81-3			+			NA	[20]
farnesyl acetate	29548-30-9			+			NA	[20]
Isopentyl propionate	105-68-0			+			No repels foraging	[12,20]
(E)-oct-2-en-1-ol acetate	2371-13-3		+				NA	[18]
phenethyl acetate	103-45-7		+				NA	[18]
**Alkane/Alkene** (Koshevnikov gland)								
decane	124-18-5			+			NA	[20]
Napthalene	91-20-3			+			NA	[20]
**Acids** (Koshevnikov gland)								
Palmitic acid	1957-10-3			+			NA	[20]
Stearic acid	1957-11-4			+			NA	[20]
**Aldehydes** (Koshevnikov gland)								
Decanal	112-31-2			+			NA	[12]
**Ketones** (Mandibular gland)								
Gamma-octanoic lactone	104-50-7			+		+	Repels foraging	[12,20]
2-heptanone	110-43-0	+					Alerts, alarm behavior	[11]

Note: We gave the Chemical Abstracts Service (CAS) number just to simplify the different names that were used in different reports. For example, isopentyl acetate was reported as isoamyl acetate, or iso-amyl acetate; (E)-2-decen-1-yl acetate was reported as 1-acetoxy-2-decene; and benzyl alcohol was reported as phenyl methanol. + present in this species, NA: no available data so far.
